# Microstructure and Mechanical Properties of Functionally Graded Materials on a Ti-6Al-4V Titanium Alloy by Laser Cladding

**DOI:** 10.3390/ma18133032

**Published:** 2025-06-26

**Authors:** Lanyi Liu, Xiaoyang Huang, Guocheng Wang, Xiaoyong Zhang, Kechao Zhou, Bingfeng Wang

**Affiliations:** 1 State Key Laboratory of Powder Metallurgy, Central South University, Changsha 410083, China; 13549643733@163.com (L.L.); zhangxiaoyong@csu.edu.cn (X.Z.); zhoukechao@csu.edu.cn (K.Z.); 2 The Powder Metallurgy Research Institute, Central South University, Changsha 410083, China; 3 School of Materials Science and Engineering, Central South University, Changsha 410083, China; hxy454924892@163.com; 4 School of Materials Science and Engineering, Anhui Polytechnic University, Wuhu 241000, China; wangguochengwgc@126.com

**Keywords:** titanium alloy, functionally graded materials, laser cladding, mechanical properties, surface modification

## Abstract

Functionally graded materials (FGMs) are fabricated on Ti-6Al-4V alloy surfaces to improve insufficient surface hardness and wear resistance. Microstructure and mechanical properties and strengthening–toughening mechanisms of FGMs were investigated. The FGM cladding layer exhibits distinct gradient differentiation, demonstrating gradient variations in the nanoindentation hardness, wear resistance, and Al/V elemental composition. Molten pool dynamics analysis reveals that Marangoni convection drives Al/V elements toward the molten pool surface, forming compositional gradients. TiN-AlN eutectic structures generated on the FGM surface enhance wear resistance. Rapid solidification enables heterogeneous nucleation for grain refinement. The irregular wavy interface morphology strengthens interfacial bonding through mechanical interlocking, dispersing impact loads and suppressing crack propagation. FGMs exhibit excellent wear resistance and impact toughness compared with Ti-6Al-4V titanium alloy. The specific wear rate is 1.17 × 10^−2^ mm^3^/(N·m), dynamic compressive strength reaches 1701.6 MPa, and impact absorption energy achieves 189.6 MJ/m^3^. This work provides theoretical guidance for the design of FGM strengthening of Ti-6Al-4V surfaces.

## 1. Introduction

Ti-6Al-4V has become a critical material in aerospace structural components [1], biomedical engineering [2,3], and marine engineering equipment [4] due to its low density, high specific strength, and excellent biocompatibility. However, its relatively low surface hardness and inferior tribological performance [5] lead to adhesive wear failure [6,7] under frictional conditions, severely limiting its engineering applications in harsh wear environments [8]. Therefore, research on surface modification technologies for titanium alloys is both crucial and urgent [9]. Among various surface engineering approaches, laser cladding technology with ceramic-phase coatings demonstrates three key advantages: formation of dense metallurgical bonding coatings [10], precise compositional control [11], and fine-grained structures [12]. These combined merits establish laser cladding as the core technical pathway to overcome surface performance limitations in titanium alloys.

The laser-clad layers exhibit superior physical metallurgical bonding with substrates, fine-grained microstructures, and high strength, leading to widespread academic applications in alloy surface hardening and wear resistance enhancement [13]. Jia et al. [14] modified Ti-6Al-4V alloy surfaces via laser cladding, fabricating (TiAl)95-xCu5Nix coatings that significantly improved wear resistance. Yu et al. [15] employed coaxial powder feeding to deposit Ti/B4C/DR40 composite coatings on shaft surfaces, producing defect-free coatings with high-quality metallurgical bonding that enhanced the wear resistance of 45 steel. Poloczek et al. [16] developed titanium carbide coatings on Inconel 625 nickel-based substrates via laser cladding by incorporating hard phases (TiC, WC, or SiC particles), which demonstrated enhanced material hardness while maintaining favorable corrosion resistance. Laser cladding demonstrates significant potential as a critical technological approach for surface modification of titanium alloys.

Mechanistic studies on laser cladding have been extensively conducted. Xu et al. [17] fabricated lightweight high-entropy alloy coatings via laser cladding, combining molecular dynamics simulations to reveal the aggregation mechanisms of Al and Ti elements. Their work demonstrated that high Ti content increased the vacancy concentration, thereby deteriorating oxidation resistance. Liu et al. [18] developed Ti-containing CoCrFeMnNi high-entropy alloy coatings on 40Cr steel surfaces to enhance tribological performance. They elucidated that increasing Ti content induced a wear mechanism transition from adhesive/abrasive wear to fatigue/abrasive wear. Hu et al. [19] implemented laser cladding with nickel alloy inter layers on low-carbon steel substrates to produce Ti-6Al-4V coatings. Their findings indicated that elevating powder feed rate and scanning speed could modify melt under cooling and preferential grain growth orientation, effectively suppressing abnormal grain growth and grain boundary segregation, and thus promoting grain refinement and equiaxed grain formation. Consequently, the synergistic interaction between the distribution of vanadium (V) and aluminum (Al) within the cladding layer and the elevated undercooling critically governs the integrated wear resistance and impact toughness of the laser-clad coating.

Despite their widespread application, conventional homogeneous coatings exhibit persistent interfacial limitations, particularly thermal stress accumulation and elemental segregation phenomena. To address these interfacial limitations, functionally graded materials (FGMs) have demonstrated significant potential in materials engineering, where their controlled composition and structure gradients enable progressive property transitions and performance enhancement. Tyagi et al. [20] investigated the Ti-Ni FGM system fabricated via additive manufacturing (AM) technology, elucidating the relationship between microstructural evolution and mechanical properties. Kulkarni et al. [21] developed AA7075 FGM coatings reinforced with micro-sized boron carbide (μB4C) particles through cold spraying, demonstrating a 46.5% improvement in wear resistance attributed to enhanced load transfer by the reinforcing phase.

In this study, FGM coatings were fabricated on Ti-6Al-4V substrates using coaxial powder feeding laser cladding with continuous wave (CW) mode. Comprehensive characterization methods were employed to analyze the mechanical properties, microstructure, and phase composition of the clad layers, with particular emphasis on the microstructural formation mechanisms. This research aims to address the challenges of low surface hardness, poor wear resistance, and insufficient impact toughness of Ti-6Al-4V titanium alloys, thereby promoting their applications in aerospace and biomedical fields.

## 2. Materials and Methods

**Figure 1:**
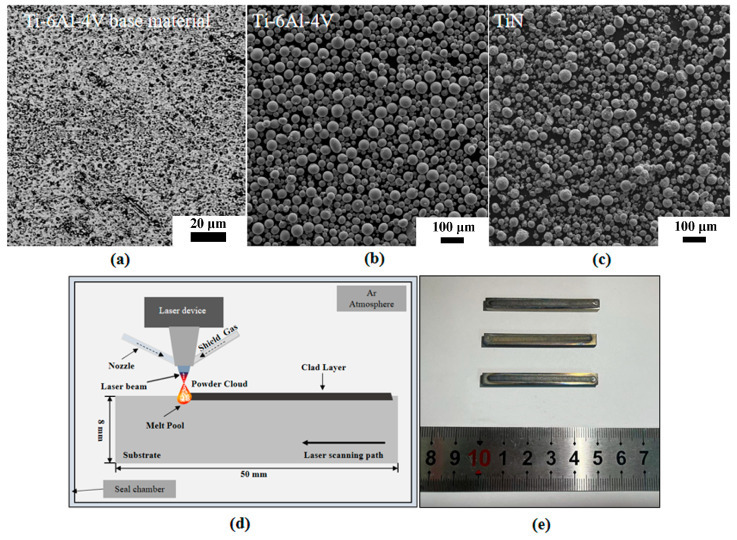
The raw materials, processes, and products of the laser cladding process. (**a**) Metallographic micrograph of the base material, (**b**,**c**) Ti-6Al-4V and TiN powder, (**d**) the laser cladding process, (**e**) photo of the FGM specimens.

The Ti-6Al-4V titanium alloy strip plate was selected as the substrate, and the base material specimen (Specimen B) served as the control group, with its initial microstructure shown in Figure 1a. Cladding materials consisted of Ti-6Al-4V and titanium nitride (TiN) spherality powders (Figure 1b and Figure 1c, respectively) supplied by QIZHI Advanced Materials Co., Ltd. (Changsha, China), featuring particle diameters ranging between 33 and 83 μm and purity exceeding 99.9%. Prior to laser cladding experiments, substrates were ground with #400 and #800 silicon carbide abrasive papers to thoroughly remove surface oxide layers and machining residues. Specimens subsequently underwent 15 minutes of ultrasonic cleaning in industrial ethanol followed by 6 hours of drying at 120 °C in a vacuum oven. This pretreatment protocol minimizes interfacial contamination effects while controllably maintaining surface roughness to mitigate specular reflection of the laser beam from overly smooth surfaces.

The laser cladding process schematic is shown in Figure 1d. A YLPM-CLAD-6000 (Tianjin Kaipulin Photoelectric Technology Co., Ltd., Tianjin, China) laser system with a maximum power of 6 KW was used to deposit cladding layers on Ti-6Al-4V substrate. To prevent oxidation of the molten pool, the entire cladding process was conducted in a custom-built argon shielding chamber (purity ≥ 99.9%, flow rate 5 L/min), with 5 min of pre-purging to ensure an argon atmosphere. During laser cladding, Ti-6Al-4V and TiN powders were separately placed in feeders, transported to the nozzle via a coaxial powder delivery system, and rapidly melted under the high-energy laser beam. The key process parameters included a laser scanning speed (V) of 10 mm/s, a laser spot diameter (d) of 3 mm, a powder feed rate of 0.5 r/min, and a carrier gas flow rate of 7 L/min. Three experimental groups were prepared: C1 and C2 employed Ti-6Al-4V powder for single-pass cladding on the intermediate layer at 1200 W and 1400 W power, respectively, while C3 utilized TiN powder for single-pass cladding on the intermediate layer at 1200 W power. Subsequently, TiN powder was deposited via single-pass cladding at 1200 W onto the intermediate layers of C1, C2, and C3, fabricating functionally graded materials (FGMs), with the actual appearance shown in Figure 1e. Critical laser cladding and remelting parameters are detailed in Table 1.

Dry sliding friction and wear performance of specimens were evaluated at room temperature using a reciprocating rotational tribometer (TRB type, CSM Instruments, Peseux, Switzerland). Both clad and unclad Ti-6Al-4V alloy plates were cut into 3 mm × 3 mm × 3 mm cubic specimens for testing. A φ50 mm × 10 mm Ti-6Al-4V disk was selected as the counterpart, with counterclockwise rotation during testing. Key parameters were set as normal load F_n_ = 5N, sliding velocity 100 cm/s, data acquisition frequency 1.0 Hz, and total wear distance d = 600 m.

The mass loss due to wear was measured using an electronic analytical balance with 0.1 mg accuracy. The wear resistance of the cladding layer was quantitatively characterized by specific wear rate (R), calculated as:

(1) R=△V/(d×Fn)

where Δ V = volume change before/after wear (mm^3^), d = total wear distance (m), and Fₙ = normal load (N). The dimensions (width, length, thickness) of specimens before/after wear were measured using a digital micrometer with 1 μm accuracy, with triplicate measurements per dimension averaged for data processing.

The nanoindentation hardness (H) and elastic modulus (E) across different cladding layer regions and heat-affected zones were evaluated using a nanoindenter (Nano Test Vantage, Micro Materials Ltd., Wrexham, UK). Four indentations were performed per region with a diamond Berkovich pyramidal indenter (tip radius 100 nm). Testing was conducted under ambient conditions with key parameters: maximum indentation load 30 mN, loading/unloading rates 5.0 mN/s, contact velocity 0.5 μm/s, and peak load holding time 40 s. Elastic modulus values were calculated via the Oliver–Pharr method [22] based on load-displacement curves obtained from nanoindentation experiments.

Metallographic specimens were prepared via wire electrical discharge machining, with cross-sectional regions of the cladding layer encapsulated in epoxy resin. After grinding/polishing, specimens were etched with Kroll’s reagent (15 s etching time) and observed under an optical microscope (OM, DM2500M, Leica Microsystems, Wetzlar, Germany) for microstructural characterization.

Detailed microstructural analysis was conducted using a field-emission scanning electron microscope (SEM, MIRA3 LMH, TESCAN, Brno, Czech Republic) equipped with energy-dispersive spectroscopy (EDS) for elemental mapping across cladding cross-sections. The phase composition of the fused cladding was characterized by X-ray diffraction (XRD, Empyrean series, Malvern Panalytical, Malvern, UK) with a 2θ range of 25–85° at a scanning rate of 5°/min (step size: 0.02°). Electron probe micro-analysis (EPMA, JXA-8230, JEOL, Tokyo, Japan) provided quantitative chemical composition data for different microstructural features.

Cylindrical specimens for dynamic mechanical analysis were fabricated from Ti-6Al-4V functionally graded materials (FGMs) via electrical discharge wire cutting (EDWC) to achieve nominal dimensions of φ6 mm × 7.2 mm. High-strain-rate compressive behavior was characterized using a split-Hopkinson pressure bar (SHPB) apparatus at ambient temperature (298 K). The derivation of true stress–strain constitutive relationships for cylindrical specimens under high-rate deformation, incorporating corrections for interfacial friction and thermal softening effects, is comprehensively delineated in the Supplementary Materials.

## 3. Results

### 3.1. Microstructure

#### 3.1.1. Optical Microscopy

**Figure 2:**
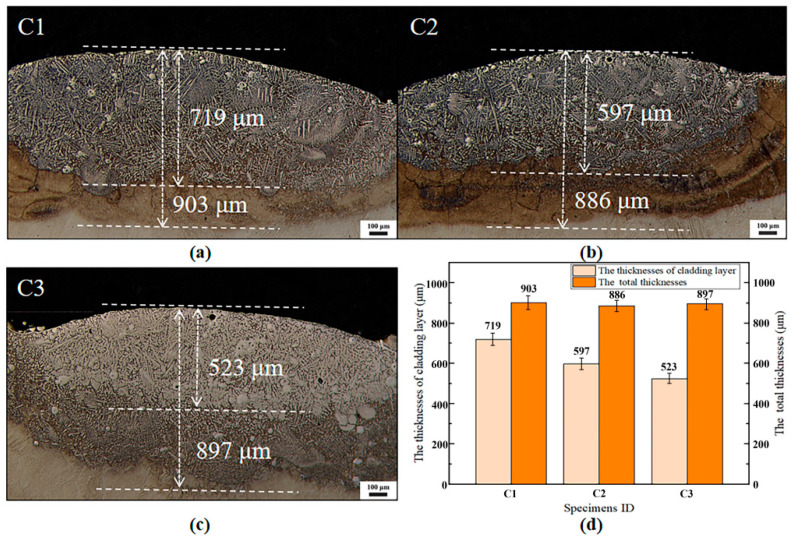
Optical micrographs of the FGMs: (**a**–**c**) the C1–C3 specimens, (**d**) the thicknesses of the FGMs’ cladding layer.

The thicknesses of the cladding layer and heat-affected zone (HAZ) of the Ti-6Al-4V titanium alloy gradient functional materials (FGMs) are clearly determined via optical microscopy. Additionally, the interlayer grain morphology and alloying degree of the FGMs are preliminary characterized, as shown in Figure 2a–c. The optical micrographs of the cladding layers for specimens C1, C2, and C3 are showed in Figure 2a–c. The cladding layers of specimens C1, C2, and C3 are dense, uniform, and thick, with no significant defects such as pores or microcracks, indicating favorable overall cladding quality in the FGMs.

Comparing the alloying degree of ceramic powders, specimen C3 (traditional double-layer TiN powder cladding) exhibits a higher number of incompletely melted TiN particles, whereas specimens C1 and C2 show fewer, suggesting that the Ti-6Al-4V powder interlayer pre-deposition facilitates the alloying process in the FGM cladding layers. Among specimens C1 and C2, specimen C1 demonstrates the highest alloying degree, with the fewest incompletely melted TiN particles and a high proportion of uniformly distributed dendrites and equiaxed grains. This implies that the alloying degree during ceramic powder laser cladding does not necessarily improve with increasing power. As shown in Figure 2d, the cladding layer thicknesses of the FGMs are 719 μm, 597 μm, and 523 μm, while the total thicknesses of the cladding layer and HAZ are 903 μm, 886 μm, and 897 μm for specimens C1, C2, and C3, respectively. The dilution ratio of the specimens can be calculated using the following formula:

(2) η=h/(H+h)

where h represents the melt depth of the substrate (μm), and H represents the height of the clad layer (μm). Based on the aforementioned formula, the dilution ratios can be calculated as η_C1_ = 0.20, η_C2_ = 0.33, and η_C3_ = 0.42. Specimen C1 achieves the highest alloying degree due to three key factors: (1) optimized laser power creating an ideal heat-affected zone (h_C1_ = 184 μm) that prevents excessive heat accumulation while ensuring sufficient energy for complete TiN dissolution, (2) uniform temperature distribution across the molten pool that maintains stable fluid dynamics and prevents localized overheating-induced TiN decomposition, and (3) effective thermal mediation by the TC4 interlayer that balances heat dissipation with melting requirements, yielding a homogeneous microstructure.

The incorporation of Ti-6Al-4V enhances the adaptability to laser parameters, allowing the FGMs to tolerate a broader laser power range and avoiding substrate overheating caused by the ultra-high power required for pure TiN. Simultaneously, the Ti-6Al-4V ductile phase alleviates stress concentration in brittle TiN through plastic deformation, reducing crack density. Meanwhile, the Ti-6Al-4V powder, with a composition consistent with the substrate material, reduces the interfacial compositional gradient and enhances the metallurgical bonding strength. The addition of Ti-6Al-4V balances the thermal expansion mismatch between TiN and the Ti-6Al-4V substrate, thereby reducing thermal stress and achieving residual stress reduction, which further minimizes crack propensity.

#### 3.1.2. Scanning Electron Microscopy

**Figure 3:**
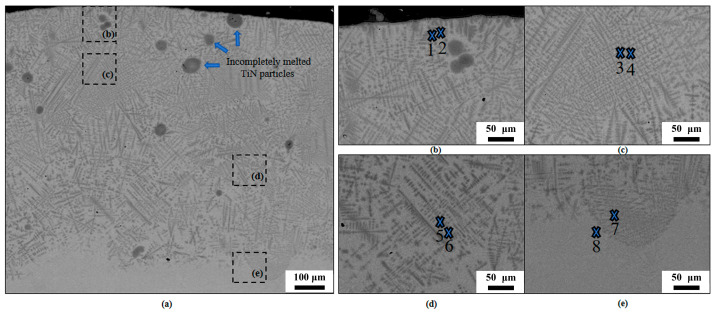
SEM micrographs of the FGM of specimen C1: (**a**) cross-sectional overview, (**b**) top layer, (**c**) middle layer, (**d**) bottom layer, (**e**) the HAZ.

Figure 3 presents SEM characterization results of the cross-sectional microstructure of the cladding layer in specimen C1 of the FGM. Figure 3b–e are magnified views corresponding to the respective regions marked in Figure 3a, representing the top layer, middle layer, bottom layer, and HAZ of the cladding layer in specimen C1.

SEM observations of specimen C1 reveal distinct gradient grain morphology in the FGMs: The top cladding layer of specimen C1 primarily consists of coarse columnar grains, with fine dendrites and numerous tiny equiaxed grains (within 0.5 μm) dispersed within inter-columnar gaps. This layer also contains a small number of incompletely melted TiN powder particles and minimal pores (within 0.3 μm). The middle layer is almost completely dense, characterized by reduced columnar grains with traces of fragmentation and an increased density of fine dendrites arranged in an orderly manner. The bottom cladding layer is pore-free, displaying fragmented columnar grains broken into regular, fine equiaxed grains, forming a mixture of fragmented columnar grains, grown dendrites, and equiaxed grains. At the boundary between the HAZ and the substrate, an irregular wavy metallurgical bonding interface is clearly observed. Above this interface, highly aligned and extremely fine dendrites are present, while below it, disordered elongated acicular martensitic α phases are distributed. The grains become progressively finer and sparser in the substrate direction.

**Figure 4:**
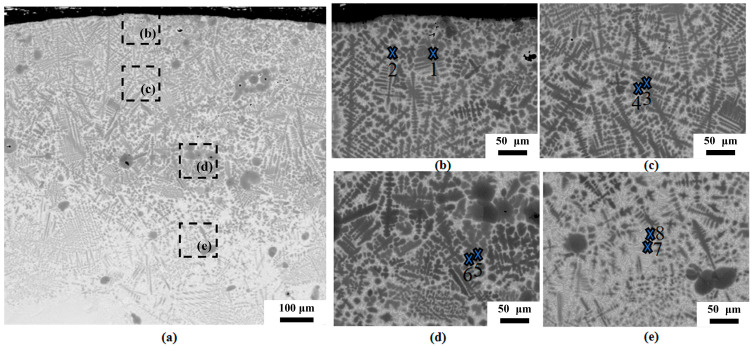
SEM micrographs of the FGM of specimen C3: (**a**) cross-sectional overview, (**b**) top layer, (**c**) middle layer, (**d**) bottom layer, (**e**) the HAZ.

Figure 4a–e exhibit the SEM microstructural characteristics of the cross-sectional cladding layer in Ti-6Al-4V functionally graded material (C3 specimen), with Figure 4b–e showing magnified views of the regions annotated in Figure 4a. Critical observations reveal a distinct interface between the cladding layer and the HAZ, along with significantly more incompletely melted TiN particles than specimen C1, densely arranged dendrites with regularly oriented secondary arms in the upper cladding region, transitional features of dendrite fragmentation toward equiaxed grain formation in the middle layer, more pronounced dendrite fragmentation accompanied by incompletely melted TiN particles in the bottom layer (in contrast to the nearly particle-free equivalent zone in C1), and predominantly equiaxed grains with increased matrix phase fraction, indicating elevated β-Ti content within the HAZ.

### 3.2. Phase Analysis and Element Distribution

**Figure 5:**
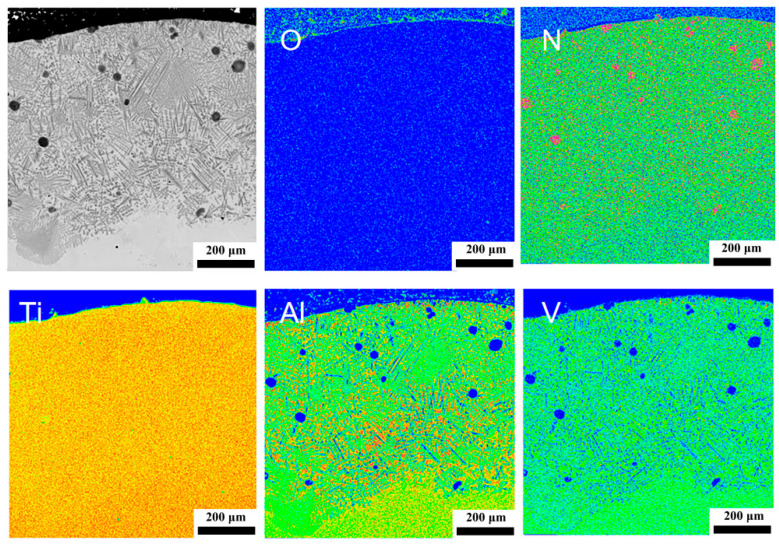
EPMA elemental mapping results of specimen C1.

Figure 5 displays the EPMA elemental mapping of O, N, Ti, Al, and V in the cladding layer of specimen C1, revealing distinct concentration gradients of Al and V. Oxygen exhibits homogeneous distribution with extremely low concentration, showing only minor surface enrichment. Nitrogen accumulates locally at the cladding surface and incompletely melted TiN particles, with higher distribution within dendrites but lower levels in interdendritic regions, where it aggregates at grain boundaries. Titanium demonstrates relatively uniform distribution, though it is slightly enriched in dendrites compared to interdendritic zones. Aluminum (originating from Ti-6Al-4V powder or substrate) is enriched at the surface and distributed in interdendritic regions, exhibiting a decreasing concentration gradient from the top to the bottom cladding layer until recovering to baseline levels near the substrate. Vanadium (similarly derived from Ti-6Al-4V powder or substrate) is predominantly segregated at dendrite boundaries with observable concentration gradients.

Figure 3a–e displays the electron probe microanalysis (EPMA) point-scanning locations for O, N, Ti, Al, and V element distributions in the cladding layer of specimen C1, with Figure 3b–e corresponding to specific regions marked in Figure 3a. Figure 3b illustrates EPMA point locations at the cladding top layer: point 1 within a dendrite core and point 2 near an interdendritic region. Similarly, Figure 3c–e represent element analysis points at the middle layer, bottom layer, and heat-affected zone (HAZ), where points 3, 5, and 7 are selected from dendrite cores and points 4, 6, and 8 from adjacent interdendritic zones.

Based on the EPMA analysis results from Table 2, titanium (Ti) exhibits uniform distribution throughout the cladding layer, though its concentration is slightly higher in dendrite trunks than in interdendritic regions; oxygen (O) shows homogeneous overall distribution but relatively higher content in dendrites at the top cladding layer; nitrogen (N) is significantly enriched within dendrite interiors, creating distinct concentration difference between dendritic and interdendritic zones; and aluminum (Al) and vanadium (V) are predominantly distributed in interdendritic regions, with their concentrations progressively decreasing along the cladding depth gradient.

Following identical point-selection methodology, the eight-point EPMA elemental analysis results in Table 3 correspond to the marked locations within the C3 cladding layer in Figure 4, providing weight percentage (wt%) data for intradendritic and interdendritic positions across the upper, middle, and lower cladding layers as well as the heat-affected zone (HAZ). Oxygen (O) content remains low, with only slightly elevated levels observed within dendrites of the upper cladding layer; titanium (Ti) demonstrates uniform distribution, exhibiting marginally higher concentrations intradendritically than interdendritically; nitrogen (N) accumulates preferentially within dendrites of the cladding layer; and aluminum (Al) and vanadium (V) are predominantly distributed in interdendritic regions.

**Figure 6:**
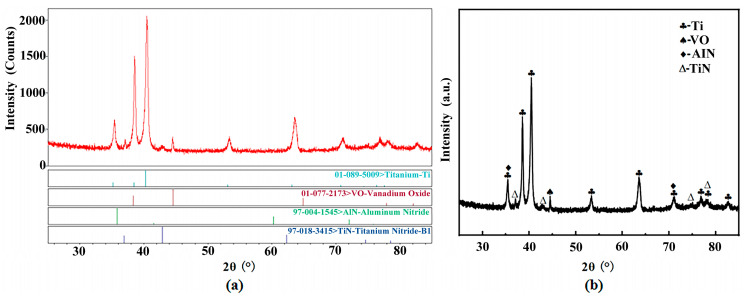
XRD results: (**a**) phase identification, (**b**) indexing results.

To investigate the formation of compounds in the C1 cladding layer, X-ray diffraction analysis was performed. Figure 5 shows the XRD analysis results of the C1 sample, with Figure 6a demonstrating the phase identification within the cladding layer. The primary phases identified at 2θ positions ranging from 35° to 45° and from 60° to 80° include Ti (PDF #01-089-5009), VO (PDF #01-077-2173), AlN (PDF #97-004-1545), and TiN (PDF #97-018-3415). Figure 6b shows the intuitive calibration results for Ti, VO, AlN, and TiN.

### 3.3. Mechanical Properties

#### 3.3.1. Nanoindentation Hardness and Elastic Modulus

**Figure 7:**
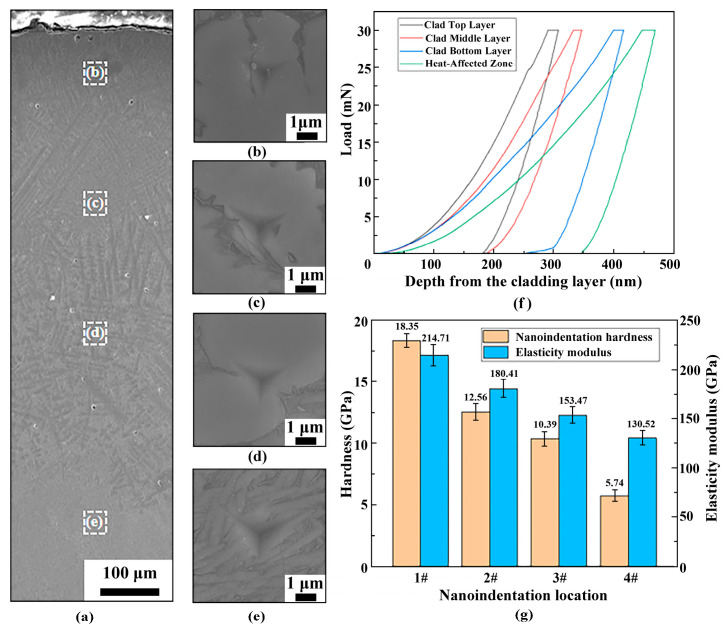
Nanoindentation results for specimen C1. (**a**) SEM images of nanoindentation locations and nanoindentations obtained at a load of 30 mN on the cross-section: (**b**) cladding top layer, (**c**) cladding middle layer, (**d**) cladding bottom layer, (**e**) heat-affected zone, (**f**) nanoindentation load-depth curves, (**g**) nanoindentation hardness and elastic modulus at different locations.

Nanoindentation testing across the cross-section of specimen C1 yielded the results shown in Figure 7. Figure 7a illustrates the positioning of nanoindentations, where the top cladding layer (Figure 7b), middle cladding layer (Figure 7c), bottom cladding layer (Figure 7d), and heat-affected zone (HAZ) (Figure 7e) correspond to the SEM images of nanoindentations obtained under a 30 mN load at locations 1#, 2#, 3#, and 4#, respectively.

Figure 7f shows the nanoindentation load–depth curves, indicating shallower indentation depths in the top cladding layer, which exhibits the highest nanoindentation hardness. Under identical loading durations, the maximum indentation depths for the top cladding layer, middle cladding layer, bottom cladding layer, and HAZ of specimen C1 are approximately 346, 416, 353, and 468 nm, respectively. Figure 7g summarizes the nanoindentation hardness (H) and elastic modulus (E) across these regions: the top cladding layer (Figure 7b), middle cladding layer (Figure 7c), bottom cladding layer (Figure 7d), and HAZ (Figure 7e) exhibit nanoindentation hardness values of 18.35, 12.56, 10.39, and 5.74 GPa, respectively, with the top cladding layer showing the highest hardness. Their elastic moduli are 214.71, 180.41, 153.47, and 130.52 GPa, respectively. The ratio of nanoindentation hardness to Young’s modulus (H/E) reflects the material’s friction and wear performance, serving as a critical parameter for wear resistance [23]. A higher H/E ratio indicates superior wear resistance. Calculation reveals that H/E_1#_ = 0.085 is the highest among the four locations, and the H/E ratio decreases gradiently with increasing distance from the surface. These results confirm that the surface hardness of the FGM specimen significantly exceeds that of the conventional Ti-6Al-4V titanium alloy, with distinct gradients in nanoindentation hardness and wear resistance, decreasing from the high hardness and wear resistance at the cladding surface toward the substrate.

#### 3.3.2. Wear Resistance

**Figure 8:**
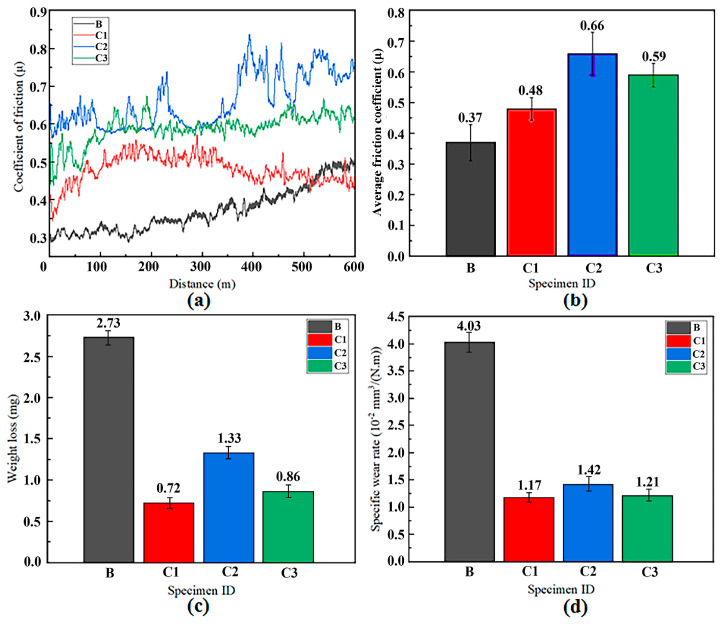
Wear resistance of Ti-6Al-4V matrix and the FGM cladding. (**a**) Curve of coefficient of friction versus distance traveled, (**b**) average coefficient of friction, (**c**) weight loss, (**d**) specific wear rate.

Figure 8 demonstrates the wear resistance of the Ti-6Al-4V titanium alloy substrate (B) and the FGMs (C1, C2, C3) at room temperature. Figure 8a displays the friction coefficient variation with sliding distance. For specimen B, the friction coefficient gradually increases and stabilizes at a higher value during sliding friction. In contrast, the FGM specimens exhibit a rapid initial rise in the friction coefficient upon contact with the counterpart, followed by fluctuations in the intermediate stage and eventual stabilization. Comparing C1 and C2 reveals that the intermediate cladding layer’s laser power significantly influences the friction coefficient of the cladding surface. Comparing C1 and C3 confirms that pre-depositing Ti-6Al-4V intermediate cladding layers reduces surface roughness and friction coefficient fluctuations in FGMs. Figure 8b presents the average friction coefficients; the substrate (B) has the lowest value (0.37μ), while the cladding specimens C1, C2, and C3 exhibit average friction coefficients of 0.48, 0.66, and 0.59 μ, respectively. Specimen C1 shows the smallest fluctuation and highest stability, whereas C2 displays the highest average friction coefficient and largest fluctuation. Figure 8c quantifies the post-wear mass loss: the substrate (B) suffers the highest loss (2.73 mg), while cladding specimens C1, C2, and C3 exhibit mass losses of 0.72, 1.33, and 0.86 mg, respectively. Specimen C1 achieves the minimal mass loss, and all cladding specimens show reduced wear compared to the substrate. Figure 8d compares the specific wear rates, with the substrate (B) demonstrating the highest value (4.03 × 10^−2^ mm^3^/(N·m)), while FGM specimens C1, C2, and C3 exhibit values of 1.17 × 10^−2^, 1.42 × 10^−2^, and 1.21 × 10^−2^ mm^3^/(N·m), respectively. Specimen C1 demonstrates the lowest specific wear rate, achieving a 3.44-fold improvement over the substrate, which confirms that the Ti-6Al-4V titanium alloy FGMs significantly enhance surface wear resistance.

To further demonstrate the enhancement in wear resistance, the specific wear rate ratios between the substrate before cladding and the optimal wear-resistant specimen (e.g., sample C1 exhibiting a 344% improvement) in this study are compared with the literature data, as summarized in Table 4. The comparative specimens include (TiAl)95-xCu5Nix coatings deposited via laser cladding on Ti–6Al–4V substrates [14], Al-µB_4_C/BNNP coatings fabricated as functionally graded materials (FGMs) by cold spraying on AA7075 aluminum substrates [21], NbMoTaWTi high-entropy alloy coatings synthesized via laser cladding on Ti–6Al–4V substrates [12], and TC4-LST + Cr coatings prepared by laser surface texturing (LST) on Ti–6Al–4V substrates [24]. Evidently, the C1 specimen demonstrates the most significant improvement in substrate surface wear resistance among these groups, surpassing both the cold-sprayed FGMs and other laser-clad or LST specimens on Ti–6Al–4V alloys.

**Figure 9:**
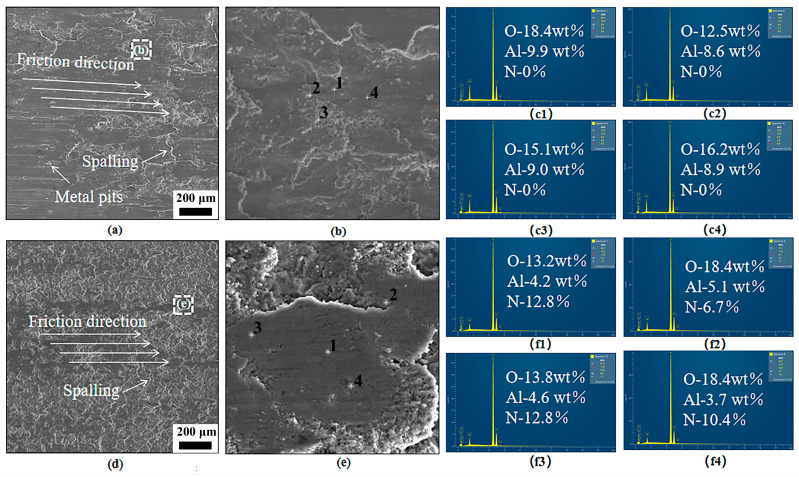
Surface SEM morphology and EDS spot-scanning results after friction wear tests for specimens B and C1: (**a**–**c**) matrix (B), (**d**–**f**) specimen C1.

To comprehensively analyze the wear-resistance mechanisms of Ti-6Al-4V functionally graded materials (FGMs), scanning electron microscopy (SEM) and energy-dispersive spectroscopy (EDS) are employed to characterize surface morphologies of specimens B (substrate) and C1, as shown in Figure 9. Figure 9a displays the worn surface SEM morphology of specimen B, with Figure 9b providing a magnified view. Numerous deep grooves and wide scratches parallel to the sliding direction are observed, accompanied by metallic debris spalling (size ≈ 30 μm) and wear pits. EDS point analysis at marked locations in Figure 9c reveals the absence of nitrogen but significant oxygen enrichment (average of 15.55 wt%), indicating thermal oxidative wear during friction. Combined observations confirm that substrate B primarily undergoes abrasive and adhesive wear mechanisms, with secondary thermal oxidation. Figure 9d presents the worn surface morphology of specimen C1, with Figure 9e showing localized details. The C1 surface exhibits improved flatness, with only minor spalling in wear zones; the absence of fatigue pits or metallic debris; and a significantly reduced groove/scratch density. EDS point analysis in Figure 9f quantifies the average elemental compositions: nitrogen (10.68 wt%), oxygen (15.95 wt%), and aluminum (4.4 wt%). Combined with the EPMA results, these data confirm the presence of TiN-AlN eutectic structures in C1’s cladding layer. The TiN hard phase resists abrasive cutting, while the AlN phase’s high thermal conductivity (320 W/m·K) dissipates frictional heat, preventing thermal softening. Consequently, C1’s wear mechanism is dominated by thermal oxidative wear with minor abrasive contributions, accompanied by limited adhesive wear.

#### 3.3.3. Impact Toughness

**Figure 10:**
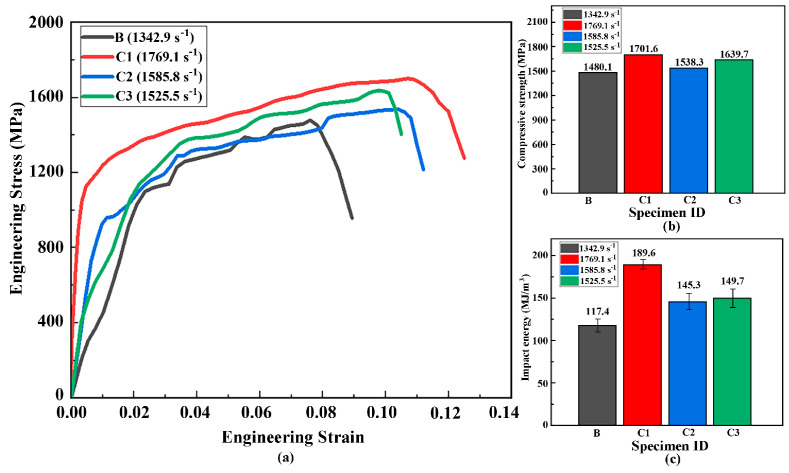
The results of SPHB tests for specimens B and C1: (**a**) The stress–strain curves of the Ti-6Al-4V titanium alloy substrate and the FGM specimens, (**b**) the dynamic compressive strength; (**c**) the impact energy absorption capacity of specimens B-C3.

Figure 10 presents the split-Hopkinson pressure bar (SHPB) test results of the Ti-6Al-4V titanium alloy matrix (B) and FGM specimens (C1, C2, C3) at room temperature. Figure 10a displays the engineering stress–strain curves of the specimens subjected to high strain rates (1342.9–1769.1 s^−1^) at room temperature. The impact energy, defined as the area under the curves in Figure 10a, evaluates the material’s energy absorption ability during high-strain-rate deformation. Figure 10b,c compare the dynamic compressive strength and impact energy absorption of B–C3. Specimen C1 exhibits optimal high-strain-rate mechanical properties at 1769.1 s^−1^, achieving a dynamic compressive strength of 1701.6 MPa and an impact energy of 189.6 MJ/m^3^. Comparing the curves of C1 and C3 reveals that under identical intermediate cladding laser power, the pre-deposited Ti-6Al-4V powder intermediate layer significantly enhances the impact resistance of FGMs. Additionally, Figure 10a demonstrates that the C1 curve has the steepest slope in the stress–strain plot, indicating the highest strain rate sensitivity, which reflects its pronounced mechanical response to loading rates.

Compared to the titanium alloy substrate (B), the FGM specimens exhibit increased dynamic compressive strength and impact energy, indicating enhanced impact toughness. The FGM structure incorporates an intermediate Ti-6Al-4V layer as a metallic binder phase. Its melting point (~1600 °C) is lower than that of TiN (2950 °C), effectively reducing the overall melting point of the cladding layer and improving molten pool flow uniformity. This optimizes molten pool behavior, facilitating the formation of titanium matrix composites with uniformly dispersed TiN particles. The hard TiN phase provides high hardness, while the Ti-6Al-4V matrix acts as a ductile phase that absorbs impact energy through dislocation entanglement. This synergy achieves concurrent enhancements in impact toughness and mechanical properties.

## 4. Discussion

### 4.1. Rapid Solidification in the FGMs

The extreme non-equilibrium cooling process (cooling rate: 10^3^–10^6^ K/s) at the molten pool edge governs the FGMs. Unlike conventional equilibrium solidification, the kinetic constraints induced by ultrahigh cooling rates force the melt to deviate from the steady-state phase transformation trajectory predicted by classical phase diagrams, resulting in unique metastable microstructures and composition distribution features. This process involves three synergistic mechanisms:

(1) Metastable phase formation and interface strengthening

The extreme non-equilibrium cooling process experienced at the molten pool edge governs the microstructural formation mechanism in functionally graded materials (FGMs). EPMA mapping (Figure 5) and EDS analysis (Figure 9f) demonstrate that the surface composition of the C1FGM specimen constitutes a Ti-N-Al multicomponent system, with XRD results (Figure 6) confirming the presence of TiN and AlN phases within the cladding layer. Under ultrahigh cooling rates, long-range diffusion of solute atoms becomes significantly suppressed. This suppression prevents the Ti-N-Al system from completing steady-state phase separation, resulting in the formation of a metastable TiN-AlN eutectic structure via a non-equilibrium eutectic reaction [25]. In this structure, the TiN hard phase resists abrasive cutting, while the AlN phase facilitates rapid heat dissipation through high thermal conductivity. Their synergy imparts ultrahigh hardness and exceptional anti-abrasive wear resistance to the material, enabling superior performance of the C1 specimen during friction and wear tests [26].

Elemental distribution data (Table 2 and Table 3) indicate a gradient decrease in Al content along the depth direction of the cladding layer. As cooling rates diminish, the microstructure undergoes progressive evolution: the surface region develops a high-volume-fraction TiN-AlN eutectic structure due to solute trapping effects, whereas the interior transitions gradually to a hybrid distribution of TiN dendrites and AlN agglomerates [27]. The hard ceramic phases (TiN, AlN) synergize with the ductile Ti-6Al-4V matrix to create a mechanically gradient-strengthened cladding architecture. This structure significantly enhances wear resistance while effectively preserving dynamic mechanical properties such as impact toughness. Progressive thermal expansion coefficient matching additionally reduces interfacial thermal stress, thereby improving bonding strength substantially.

(2) Grain refinement and defect regulation

High undercooling during rapid solidification significantly reduces the critical driving force for nucleation, effectively activating pre-existing heterogeneous nucleation sites (e.g., incompletely melted TiN particles, oxide inclusions). The exponential increase in nucleation density refines α-Ti matrix grains to submicron scales. Dual strengthening effects arise from grain refinement: Elevated grain boundary density hinders dislocation slip via the Hall–Petch mechanism, and nanoscale grains promote a gradual transition in deformation mechanisms from dislocation-dominated slip to grain boundary sliding, enhancing plastic compatibility. Additionally, high-density dislocation networks generated during rapid solidification amplify strain-hardening potential [28].

(3) Solute trapping and cluster pinning

The results in Table 2 and Table 3 reveal preferential segregation of Al and V within interdendritic regions, with concomitant depletion in dendritic trunks. Remarkably, for specimen C1, while the elemental sources reside in the middle clad layer/substrate, peak concentrations of Al and V occur at the uppermost clad layer, exhibiting progressive downward concentration gradients with increasing clad depth. This phenomenon is governed by Marangoni convection effects [29], wherein surface tension gradients drive upward transport of partially dissolved Ti-6Al-4V powders and substrate material toward the molten pool surface during laser cladding. Subsequent directional migration of Al/V atoms proceeds via Fick’s second law and thermal diffusion mechanisms, ultimately establishing top-down elemental concentration gradients throughout the clad layer. Nevertheless, specimen C2 lacks the Ti-6Al-4V powder interlayer, restricting Al and V derivation exclusively to the molten titanium alloy substrate. Consequently, these elements attain maximum concentrations at the intermediate clad layer through melt pool convection and diffusion-limited transport.

Anomalous solute partitioning during non-equilibrium solidification causes significant deviation in the equilibrium partition coefficient of vanadium and other alloying elements at solid–liquid interfaces. This kinetic segregation selectively enriches V atoms within interdendritic regions and along grain boundaries, as demonstrated in Table 2 and Table 3. The resulting vanadium-rich zones form V-Ti intermetallic clusters through short-range diffusion [30]. These nanoclusters enhance material performance through dual reinforcement mechanisms: firstly, they act as impenetrable barriers restricting dislocation movement, forcing dislocations to bypass clusters via bowing mechanisms and substantially increasing the critical shear stress required for dislocation multiplication [31]; secondly, their selective segregation at grain boundaries reduces boundary migration driving forces while suppressing abnormal grain coarsening at elevated temperatures through Zener pinning effects [32]. This integrated action ultimately achieves simultaneous enhancement of strength and toughness in functionally graded materials.

### 4.2. Interface Morphology of the FGMs

The non-planar wavy morphology at the bottom interface of the cladding layer fundamentally reflects the dynamic response behavior under multiphysics coupling within the molten pool. This phenomenon originates from the Marangoni convection effect driven by the temperature-dependent surface tension of liquid metal [12]: When the melt in the central region (peak temperature gradient zone) experiences significant surface tension reduction due to high temperatures, thermocapillary effects induce a directional outward flow from the center to the periphery. This flow generates toroidal convection vortices within the molten pool. The vortices create periodic disturbance waves at the molten pool front, with wavelengths exhibiting nonlinear correlations with pool oscillation frequencies [12]. The competition between melt flow and solid-phase growth destabilizes the solidification interface. When the solidification rate exceeds a critical threshold, coupling between solute redistribution and fluid perturbations triggers a transition from planar to dendritic growth. In titanium alloys, low thermal conductivity and high solidification shrinkage enhance stress-induced interface modulation, forming irregular wavy features.

The non-planar interface structure significantly enhances bonding performance in heterogeneous material systems through multiscale mechanical coupling effects: Mechanical interlocking at the wavy interface forces crack propagation paths to undergo periodic deflections [12]. This subjects crack tips to alternating mode II (shear) and mode III (tearing) stress states, substantially increasing energy dissipation during crack propagation [33]. Compared to flat interfaces, the wavy morphology reduces stress concentration coefficients while improving interfacial fracture toughness [29]. Additionally, residual compressive stress fields induced by the irregular wavy structure suppress fatigue crack initiation, endowing functionally graded materials with exceptional thermal shock resistance and high-cycle fatigue life.

### 4.3. Microstructure Mechanisms for the FGMs

**Figure 11:**
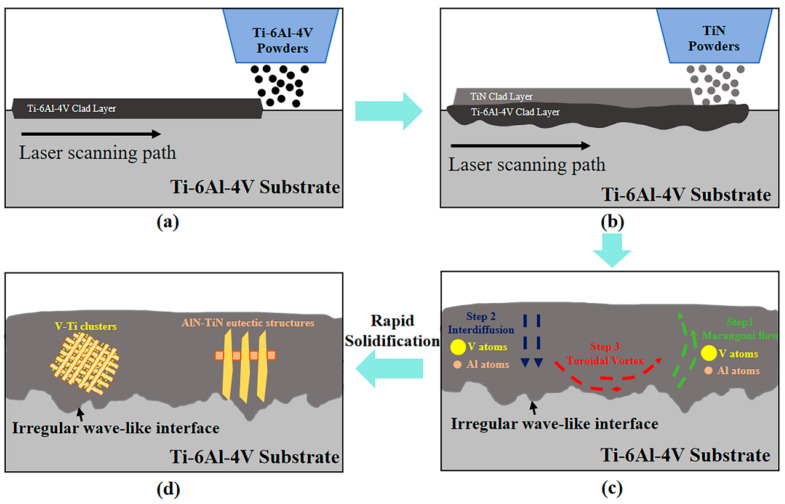
Schematic diagram of the formation mechanism of the FGM. (**a**–**d**) correspond to the four steps in the FGM formation process, respectively.

Based on the above results and analysis, it is concluded that the microstructural evolution of the FGMs fundamentally results from the alternating dominance of dynamic equilibrium and non-equilibrium mechanisms. This process is driven by the multi-field coupling of thermodynamic, hydrodynamic, and chemical interactions during the laser cladding process. Temperature gradients drive fluid flow, while fluid shear stresses influence thermal transport effects; temperature fields regulate solute diffusion rates and reaction kinetics; mechanical stirring enhances elemental mixing; and stress concentration zones accelerate interfacial reactions. As illustrated in Figure 11a–d, this process comprises four key stages, each governed by specific physical mechanisms driving the self-organized construction of compositional gradients and multi-scale structures.

Stage 1: Synergistic growth of initial cladding interface (Figure 11a). The first Ti-6Al-4V cladding layer forms through the coordinated laser energy input and substrate thermal response. Under the high energy density of the laser beam, the substrate surface and Ti-6Al-4V powder rapidly form a molten pool. The molten Ti-6Al-4V alloy undergoes instantaneous liquid flow and solidification. Due to identical chemical compositions between the cladding layer and substrate, no diffusion barrier forms at the interface. Instead, β-Ti phase epitaxial growth achieves atomic-scale metallurgical bonding: molten metal solidifies rapidly, with β-Ti phases (body-centered cubic) on the substrate surface acting as heterogeneous nucleation sites, guiding epitaxial growth of liquid metal aligned with the substrate lattice. This crystallographic continuity eliminates lattice mismatch at the interface, preventing dislocation or pore formation typical in conventional heterogeneous interfaces. Concurrently, rapid solidification suppresses grain coarsening, forming fine α-phase dendrites whose orientation aligns with molten pool thermal flow, providing a low-defect interfacial foundation for subsequent gradient structures.

Stage 2: Coupled transport in non-equilibrium molten pool (Figure 11b). With the addition of TiN powder, the molten pool exhibits distinct non-equilibrium thermodynamic characteristics. Superimposed axial and radial temperature gradients generate surface tension gradients at the molten pool surface, inducing thermocapillary convection (Marangoni effect). The resulting vortical flow dynamically mixes incompletely melted TiN particles with the molten matrix. Concurrently, solute transport involves two competing mechanisms: Fickian diffusion (Fick’s Second Law) dominates elemental intermixing of Ti, Al, and V, while thermal diffusion (Soret effect) drives directional migration of Al and V under temperature gradients. This competition between temperature-driven solute migration and concentration-driven diffusion establishes a vertical Al/V content gradient in the mid-cladding layer.

Stage 3: Dynamic evolution of gradient interface and elemental segregation (Figure 11c). Synergistic mechanical stirring and temperature gradients induce stratified molten pool flow. Mid-layer molten Ti-6Al-4V powder migrates upward due to thermocapillary convection, while incompletely melted TiN particles settle downward via density differences. Al preferentially enriches in interdendritic liquid phases due to higher solid–liquid partition coefficients, while V segregates at α-Ti grain boundaries via grain boundary adsorption. Rapid solidification “freezes” these gradient compositions into the solidified microstructure.

Stage 4: In situ microstructural self-organization (Figure 11d). During final solidification, microstructure formation is governed by local solute concentration and interfacial energy. Al-rich liquid phases undergo eutectic reactions in interdendritic regions, forming TiN-AlN lamellar structures with spacings controlled by localized cooling rates. V-Ti clusters form at grain boundaries via Ostwald ripening, with their size distributions correlating strongly with temperature gradients in the cladding layer.

The sequential evolution ultimately forms a three-level gradient architecture: at the macroscale, the TiN volume fraction progressively increases toward the cladding surface, enhancing the overall hardness of FGMs through geometric hardening effects; at the microscale, the Al concentration gradient in the α-Ti matrix and the TiN-AlN lamellar structures improve toughness via crack branching and deflection; and at the nanoscale, V-Ti clusters strengthen the material by pinning dislocations and hindering grain boundary sliding. The coupling of these cross-scale structures enables continuous transitions in mechanical properties of FGMs while avoiding interfacial failure risks inherent to traditional layered materials.

## 5. Conclusions

FGMs with a thickness of about 719 μm are successfully fabricated on a Ti-6Al-4V titanium alloy substrate. Compared to the Ti-6Al-4V substrate, the Ti-6Al-4V functionally graded materials (FGMs) exhibit significantly enhanced surface hardness, wear resistance, and impact resistance. Specimen C1 demonstrates a specific wear rate of 1.17 × 10^−2^ mm^3^/(N·m), with the nanoindentation hardness and elastic modulus of the cladding top layer reaching about 18.35 GPa and 214.71 GPa, respectively. Its dynamic compressive strength and impact energy absorption are about 1701.6 MPa and 189.6 MJ/m^3^, respectively. These superior mechanical properties suggest promising applications in aerospace engine blades and biomedical implants, where combined wear and impact resistance is critical.

The FGM cladding layer exhibits the compositional gradient of Al and V elements decreasing from the surface toward the substrate. Both hardness and wear resistance of the FGM cladding layer demonstrate a gradient decline trend with increasing cladding depth. The surface layer of the FGM cladding consists of coarse columnar grains and fine TiN-AlN eutectic structures. The middle layer is composed of a hybrid microstructure of micron-sized TiN and AlN particles alongside V-Ti clusters, while the bottom layer exhibits an irregular wavy metallurgical bonding interface. The heat-affected zone is characterized by a densely packed acicular α-phase.

The microstructure evolution of FGMs involves four stages: the synergistic growth of the initial cladding interface, coupled transport in a non-equilibrium molten pool, the dynamic evolution of the gradient interface and elemental segregation, and in situ microstructural self-organization. Compared to conventional cladding materials, FGMs achieve progressive thermal expansion coefficient matching that eliminates interfacial thermal stresses. The multi-physics self-organization characteristics inherent in the graded structure mitigate interface failure risks caused by abrupt compositional transitions, thereby enabling synergistic optimization of wear resistance, impact toughness, and interfacial bonding strength.

## Figures and Tables

**Table 1:** The key process parameters of laser cladding.

Specimen ID	Laser Power (W)	Powder for the Mid-Layer	Laser Power (W)	Powder for the Outer Layer
C1	1200	Ti-6Al-4V	1200	TiN
C2	1400	Ti-6Al-4V	1200	TiN
C3	1200	TiN	1200	TiN

**Table 2:** Mass fraction of elemental analyses at different locations of EPMA points in specimen C1.

	Data	O (wt%)	Ti (wt%)	Al (wt%)	V (wt%)	N (wt%)
1	C1-1	2.57	83.482	0.814	1.34	12.309
2	C1-2	0.52	78.601	8.809	7.044	3.424
3	C1-3	0.49	85.596	0.398	1.249	13.873
4	C1-4	0.964	80.938	7.232	6.654	3.818
5	C1-5	1.275	84.838	0.619	1.271	12.969
6	C1-6	0.213	82.381	7.068	5.436	5.734
7	C1-7	0.652	85.212	0.813	1.258	12.65
8	C1-8	0.435	84.801	5.521	4.086	5.221

**Table 3:** Mass fraction of elemental analyses at different locations of EPMA points in specimen C3.

	Data	O (wt%)	Ti (wt%)	Al (wt%)	V (wt%)	N (wt%)
1	C3-1	2.345	82.906	0.114	1.161	16.021
2	C3-2	0	80.156	6.897	5.061	4.714
3	C3-3	0	83.172	0.118	1.092	15.372
4	C3-4	0.603	81.475	6.725	5.829	3.88
5	C3-5	0	83.238	0.125	1.111	16.423
6	C3-6	1.142	82.601	6.749	5.821	6.535
7	C3-7	0.286	83.829	5.807	3.915	6.704
8	C3-8	0.455	84.12	5.689	5.68	4.012

**Table 4:** Specific wear rate ratios between optimal wear-resistant coatings and substrates.

Optimal Wear-Resistant Specimen in Respective Studies	Wear Resistance Improvement
C1 FGM coating	344%
(TiAl)95-xCu5Nix coatings [14]	47%
Al-µB4C/BNNP Coatings [21]	214%
NbMoTaWTi high entropy alloy coating [12]	122%
TC4-LST + Cr coating [24]	227%

## Data Availability

The original contributions presented in this study are included in the article/Supplementary Material. Further inquiries can be directed to the corresponding author.

## Supplementary Materials

The following supporting information can be downloaded at: https://www.mdpi.com/article/10.3390/ma18133032/s1, Calculation of dynamic mechanical parameters on a split Hopkinson pressure bar (SHPB). Reference [34] is cited in the Supplementary Materials.
