# Supplementary material for: Microstructure and Mechanical Properties of Functionally Graded Materials on a Ti-6Al-4V Titanium Alloy by Laser Cladding
Source: Materials (Basel). 2025 Jun 26;18(13):3032. doi: 10.3390/ma18133032 (PMC12250608; doi:10.3390/ma18133032)
Supplement: Supplementary file 1 [file materials-18-03032-s001.zip › materials-3679841-supplementary.pdf]

Supplementary Materials. Calculation of dynamic mechanical parameters on a split Hopkinson pressure bar (SHPB)

The SHPB is used as a loading device, and the specimens are placed between the incident bar and the transmission bar. By processing the collected electrical signal, the force between the bar and the specimen, the relative displacement between the two bars, the variation curve of the specimen reduction and the force can be obtained. The bullet fired by the air gun strikes the incident rod to obtain the incident wave. The specimen deforms at a high speed under the action of incident pulse loading, and the reflected wave is generated in the incident rod. The transmission wave is produced in the transmission rod. The strain signals of the incident, reflected, and transmitted waves can be received and recorded by the strain gauge.

The stress, strain rate, true strain, and true stress of the specimen during dynamic loading and deformation can be calculated by the following formula[1]:

$$\sigma = \frac{E_0 A_s \varepsilon_t(t)}{A_0} \tag{1}$$

$$\dot{\varepsilon} = \frac{2C_0}{L}[\varepsilon_i(t) - \varepsilon_t(t)] \tag{2}$$

$$\varepsilon = \frac{2C_0}{L}\int_0^t [\varepsilon_i(t) - \varepsilon_t(t)]dt \tag{3}$$

$$\varepsilon_T = \ln(1 + \varepsilon) \tag{4}$$

$$\sigma_T = \sigma(1 + \varepsilon) \tag{5}$$

where σ, ε, $\dot{\varepsilon}$, ε_t, and σ_T are the stress, strain, strain-rate, true stress, and true strain, respectively; εi(t) and εt(t) represent voltages from the incident bar and the transmitted bar, respectively; E_0 and C_0 are the elastic modulus and elastic wave speed in the SHPB, respectively; A_s and A_0 are the cross-sectional area of the bar and the

specimens, respectively; L is the height of the specimen.

Reference

[34] M.F. Omar, H. Md Akil, Z.A. Ahmad, A.A.M. Mazuki, T. Yokoyama, Dynamic properties of pultruded natural fibre reinforced composites using Split Hopkinson Pressure Bar technique, *Materials & Design* **2010**, 31, 4209-4218. https://doi.org/10.1016/j.matdes.2010.04.036
